# Senescence and early-life performance as predictors of lifespan in a solitary bee

**DOI:** 10.1098/rspb.2024.2637

**Published:** 2025-04-16

**Authors:** Andre Szejner-Sigal, Joseph P. Rinehart, Julia Bowsher, Kendra J. Greenlee

**Affiliations:** ^1^ Biological Sciences Department, North Dakota State University, Fargo, ND, USA; ^2^ US Department of Agriculture, Fargo, ND, USA

**Keywords:** longevity, senescence, biological age, performance

## Abstract

Performance tends to decline with age, including muscle function and stress tolerance. Yet, performance can vary widely among individuals within the same age group, showing that chronological age does not always represent biological age. To better understand ageing, we need to examine what drives some individuals to age faster than others. In order to achieve this, first we need to be able to predict whether an individual will have a long or short lifespan. In this study, we conducted a longitudinal study tracking individual-level locomotor activity, chill-coma recovery time, and metabolic rates, and assessed whether early-life performance is linked to lifespan using the solitary bee *Megachile rotundata*. We found that locomotor activity and chill-coma recovery times decline in old adults. However, resting metabolic rate did not change with age. We also found low cold tolerance and low mass at emergence in early-life are linked to shorter female lifespans, showing that early-life performance can explain some of the variation in lifespan in a population. Finally, these results also show that not all traits decline with age within the same species, and shed new light on sexual dimorphism in physiological traits and ageing.

## 1. Introduction

Fitness and performance usually decline with age for most organisms [1–3]. However, lifespan and ageing rates can vary widely among individuals within a population and across species [4]. Variation in ageing between individuals of the same age group may be revealed by physiological and molecular markers of ageing more accurately than chronological age. Thus, understanding the sources of variation in the ageing process, especially within the same age groups, is essential to evolutionary biology.

Ageing is the gradual deterioration of physiological processes that drive a decline in performance, eventually resulting in death [2]. Ageing hypotheses propose a number of cellular and molecular mechanisms contributing to the ageing process, such as oxidative damage and accumulation of mutations [4,5]. Irrespective of the mechanisms, a suite of performance traits and behaviours usually declines relative to early-life levels, including muscle function, stress tolerance and energetics [6–9]. However, chronological age is not always a reliable predictor of performance, as demonstrated by widespread variation in performance within the same age group and variation in lifespan within a species. Whether an individual’s early-life performance is associated with their lifespan remains underexplored, especially non-destructive metrics that permit longitudinal studies. Linking early-life performance to lifespan has enormous potential for improving population models and conservation efforts when assessing population health.

Variation in lifespans and in performance within the same age groups are modulated by intrinsic and extrinsic factors. Lifespan can be modulated by multiple factors such as ‘ageing genes’ [10], telomere shortening [11,12] and stress exposure and tolerance [13]. Similarly, linking variation in performance within the same age group and their age at death remains an exciting avenue of research [14]. Any study that systematically utilizes same-age individuals offers insights into the extent of variation in performance occurring in that particular age and study system. However, relatively few studies continue to assess whether the observed variation is associated with ageing rates. The link between variation in performance for same-age individuals and their ageing rates may offer insights into potential targets of selection resulting in variation in lifespans.

How performance changes with age at the individual level presents a unique set of challenges. Measures of performance that are sensitive to ageing may be difficult to identify and are system-specific [3,4]. Also, studies on how performance changes with age are ideally done in longitudinal studies [15]. However, such studies are often time-consuming, resource-intensive and not always possible due to the life history of the study system (e.g. scale of lifetime, organism size and rarity). Insect study systems can overcome several of these challenges due to their relatively short lifespans and impressive diversity in life-history traits and strategies. This diversity offers a great opportunity to explore the mechanisms driving ageing and the evolution of lifespans. In this study, we use the solitary bee, *Megachile rotundata*, a well-studied organism with a relatively short adult lifespan. Bees belong to the order Hymenoptera, a group of insects with a remarkable diversity in life-history strategies and adult lifespans that range from a few days to several decades.

Here, we conducted a longitudinal study to examine how multiple performance traits (cold tolerance, locomotor activity and resting metabolic rate) change with age in adult bees. We chose these performance metrics due to their physiological importance for survival. Cold tolerance and metabolic rates are metrics of physiological robustness (i.e. an organism’s ability to maintain stable physiological functions despite external or internal disturbances and return to homeostasis) [16,17], and locomotor activity is a proxy for key behaviours such as foraging and mate finding. We hypothesized that all measured performance traits would decline through the course of the adult bee’s life. We then assessed whether early-life performance can be used as a predictor of adult lifespan, and whether long- and short-lived individuals showed specific performance patterns early in their adult life. We hypothesized that early-life cold tolerance is a better predictor of lifespan, as it is a more robust proxy of physiological condition compared with resting metabolic rates and locomotor activity. This longitudinal study, with repeated measures from adult emergence to death, provided a unique opportunity to examine how some individuals aged faster than others and identified key early-life traits associated with lifespan in a pollinator.

## 2. Methods

### (a) Insect rearing

Overwintering alfalfa leafcutting bees (*M. rotundata*) were sourced from JWM Leafcutters (Nampa, ID, USA) in March of 2023 and kept at 6°C in complete darkness until development was induced for the experiments. To induce development, brood cells were placed at 29–24°C and 16L:8D until emerging as adults. Adult bees were collected the day they emerged and individually marked using oil-based paint markers (Sharpie, IL, USA) to identify them throughout their life. Groups of 10 bees were kept in housing boxes (9 cm × 9 cm × 3 cm) on a long-day cycle at 29–24°C and 16L:8D (Percival Scientific, IA, USA) and assigned to one of the three performance experiments: cold tolerance, locomotor activity or metabolic rate. Bees were provided 50% sugar solution (Pro-Sweet, MN, USA), which was replaced twice a week. Bees had 24 h to settle in the housing boxes before performance experiments began. Throughout the experiment, bee mortality was recorded three times a week, and dead bees were removed from the boxes.

### (b) Metabolic rates

We measured oxygen consumption (VO_2_) and carbon dioxide production (VCO_2_) weekly as a proxy for resting metabolic rates of bees across their lifespan using stop-flow respirometry. Marked bees were put into 5 ml syringes and acclimated to both the syringe chamber and room temperature (22°C) for 30 min, at which point bees were at rest. Syringes were flushed with dry, CO_2_-free air, using a Drierite–Ascarite–Drierite column at a flow rate of approximately 180 ml min^−1^ and then sealed for 32 min before gas measurements. Gas samples were then injected and measured with O_2_ and CO_2_ gas analysers (Foxbox, Sable Systems International, NV, USA) at a flow rate of approximately 180 ml min^−1^ on a background of dry, CO_2_-free air, with baseline recordings in between each sample and a blank after every 30 measurements. Respirometry data were corrected for sensor drift and noise using baselines and Catmull–Rom and Savitzky–Golay filter with a 15-step window (ExpeData, Sable Systems International). Bee mass was recorded immediately after measurement (XS105 microbalance (±1 μg), Mettler Toledo, OH, USA). Respiratory exchange ratios (RER), a metric widely used to indicate the primary metabolic fuel use, were calculated for each age group as


$$RER=\dot{V}CO_{2}/\dot{V}O_{2}$$

where $\dot{V}CO_{2}$ and $\dot{V}O_{2}$ are the respective gas production rate at 22°C. Individual bees were repeatedly measured once a week until death, for a total of 120 bees, allowing individuals to fully recover for a week between repeated measurements to recover from handling stress. No bees died during the measurements.

### (c) Cold tolerance assays

To assess individual-level variation in cold stress tolerance across lifespan, we conducted chill-coma recovery time (CCRT) assays, where higher recovery times indicate low cold tolerance. In a pilot study, we identified that 45 min at 0°C was sufficient to induce a chill-coma response that most bees recovered from within 20 min (pilot data not shown). To determine CCRT, we transferred groups of adult bees (from each housing box) into 15 ml conical tubes with a cotton stopper and placed the conical tubes in an ice–water slurry at 0°C for 45 min. After the cold exposure, using a paintbrush, bees were immediately placed on their backs on a filter paper arena (10 cm in diameter). We recorded each bee’s ID and recovery time for up to 20 min at room temperature (22°C). Recovery was scored when a bee was able to fully right itself without any stimulus. Bees that did not recover in 20 min were recorded as ‘unrecovered’ and were censored in the statistical analysis. We repeated this for each individual once a week until death for a total of 140 bees, allowing individuals to fully recover for a week between repeated stress. Only two bees died during the cold exposure.

### (d) Locomotor activity

To assess individual-level variation in locomotor performance through time, we measured bee locomotor activity using protocols for this study system [18]. Briefly, we loaded individual bees into 5 ml chambers and placed those into a TriKinetics Drosophila Activity System (DAMS, MA, USA) within an incubator set at 29°C. Individual bee activity was recorded every minute for 4 h from 10.00 to 14.00, the peak locomotor activity for this species (J. B. Pithan 2023, unpublished data), and the sum was used as each bee’s total activity at each age. Individual bee activity was repeatedly measured once a week until death, for a total of 199 bees.

### (e) Statistics

All statistical analyses were performed in R v.4.2.2 [19], and data exploration was conducted according to Zuur *et al.* [20]. In all analyses, we conducted separate models for males and females due to the strong sexual dimorphism in lifespan. To assess survival across experiments, we used Cox proportional hazard models for each sex. We fitted linear mixed models for each sex using the *lme4* package, describing performance as a function of age and lifespan (i.e. age at death), with bee ID as random effects. We did model comparison and selection using Akaike information criterion to determine the best fit, including models with performance transformed with square root or natural log when it normalized the error distributions (electronic supplementary material). We included housing box ID as a random effect when it improved the model. For the metabolic rate dataset, we took the residuals of log–log transformed metabolic rate and mass, as they strongly covaried. The metabolic rate–mass residuals take into account the nonlinear relationship between mass and metabolic rate, allowing for the assessment of mass-specific metabolic rate. When assessing RER, we excluded values above 2.5 (*n* = 3) due to VO_2_ values smaller than signal-to-noise ratio from the instrument, and high values due to bees not being at rest during the trial (1.8% of data points). For CCRT, we used Cox proportional hazard models for each sex to account for censoring of individuals who did not recover within the experiment trials.

Finally, we used two approaches to determine how lifespan is associated with early-life performance. We first compared long-lived and short-lived individuals by selecting the individuals at the upper and lower 20% lifespan for each sex and experiment after centring for lifespan. This resulted in approximately 10–20 individuals for each long-lived and short-lived group and each performance experiment. These data were analysed for each sex during early life (Day 1, Day 7 and Day 14) using linear mixed models describing lifespan as a function of age, lifespan and whether they were long-lived or not (termed ‘Long.Lived’), with bee ID as random effects. For mass at emergence, we used one-way analysis of variance, as the error distribution did not violate normality. The second approach included linear regressions to assess whole age-group population and performance trends for Day 1, Day 7 and Day 14 to examine how lifespan correlates with performance.

## 3. Results

### (a) Survival

**Figure 1.**
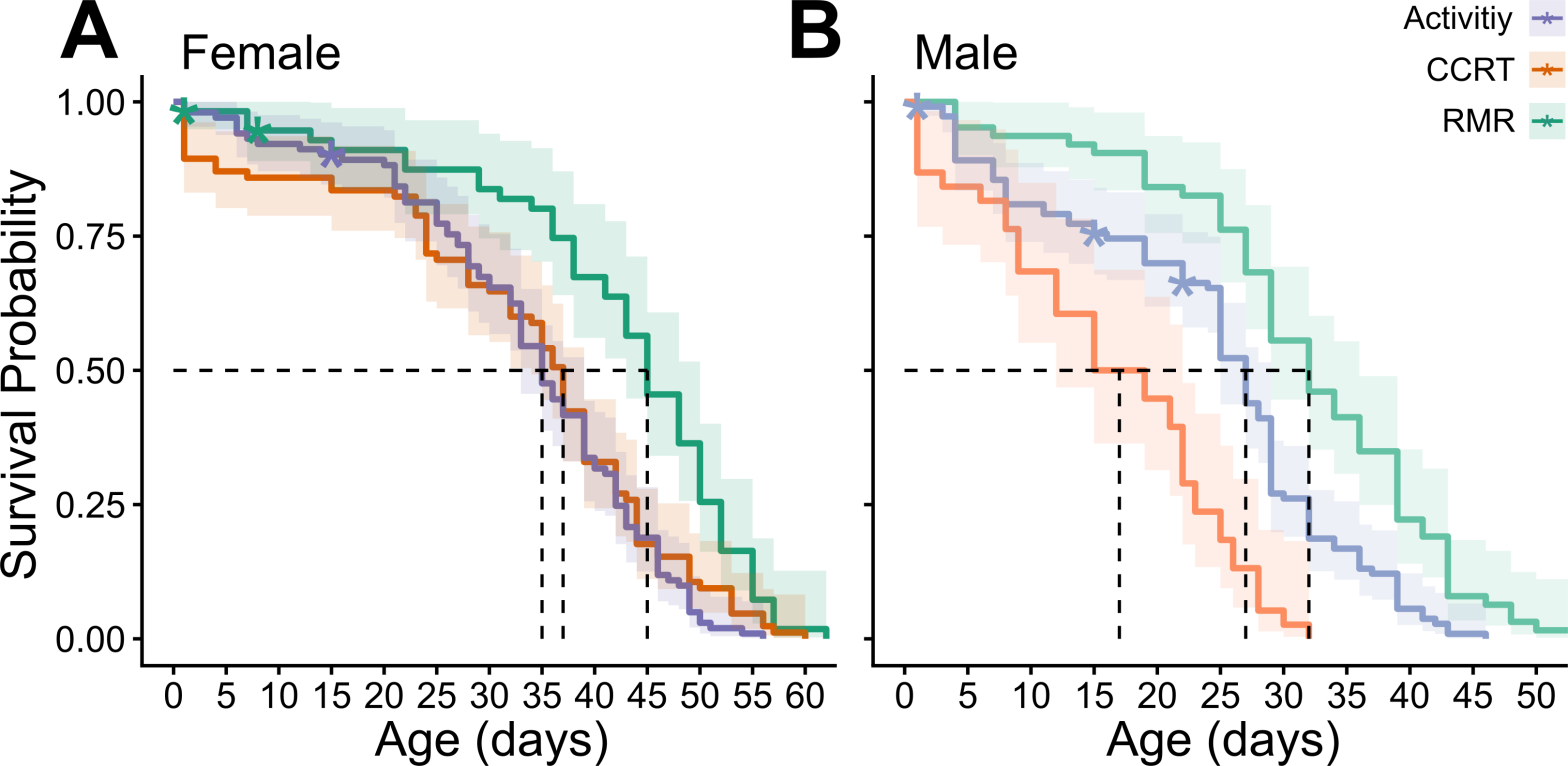
Survival curves of *Megachile rotundata* adult females (A) and males (B) for performance experiments. Dotted lines mark the median lifespan, shaded region shows the 95% CI and asterisks indicate censored individuals.

Survival curves were distinct between sexes and experiment (table 1). Metabolic rate (MR) experiments resulted in the longest lifespan for both males and females (figure 1) and had similar longevities to control treatments from past studies [21]. Female lifespan was not significantly different under locomotor activity and CCRT experiments (figure 1A). Repeated CCRT experiments had the largest effect, decreasing lifespan in male bees by nearly 50% (figure 1B).

**Table 1.** Proportional hazard models for lifespan in both female and male *Megachile rotundata* bees between experiments. CCRT, chill-coma recovery time; RMR, resting metabolic rate; HR, hazard ratio; CI, confidence interval.

experiment	HR	95% **CI**	*p* value
**female**			
RMR	—	—	
activity	1.87	1.33, 2.64	<0.001
CCRT	2.25	1.60, 3.17	<0.001
**male**			
RMR	—	—	
activity	2.07	1.49, 2.88	<0.001
CCRT	5.7	3.63, 8.97	<0.001

### (b) Metabolic rate does not decline with age

Female bees were significantly larger than male bees at emergence (*F*_1,114_ = 47.2, *p* < 0.01). Mass increased with age for males but not for females (electronic supplementary material, table S1A, figure S1A), and mass had a significant effect on resting metabolic rates (*F*_2,631_ = 70.76, *p* < 0.001; electronic supplementary material, figure 1SB). Mass-corrected metabolic rates were not significantly different across age or sex (electronic supplementary material, table S1B, figure S2). RER for male bees started at 0.8 on Day 1 and increased to approximately 1 across the rest of their lifetime, while female bees consistently maintain a RER of approximately 1 through their adult lifespan (*F*_3,599_ = 56.79, age × sex interaction *p* < 0.001; electronic supplementary material, figure S3).

**Figure 2.**
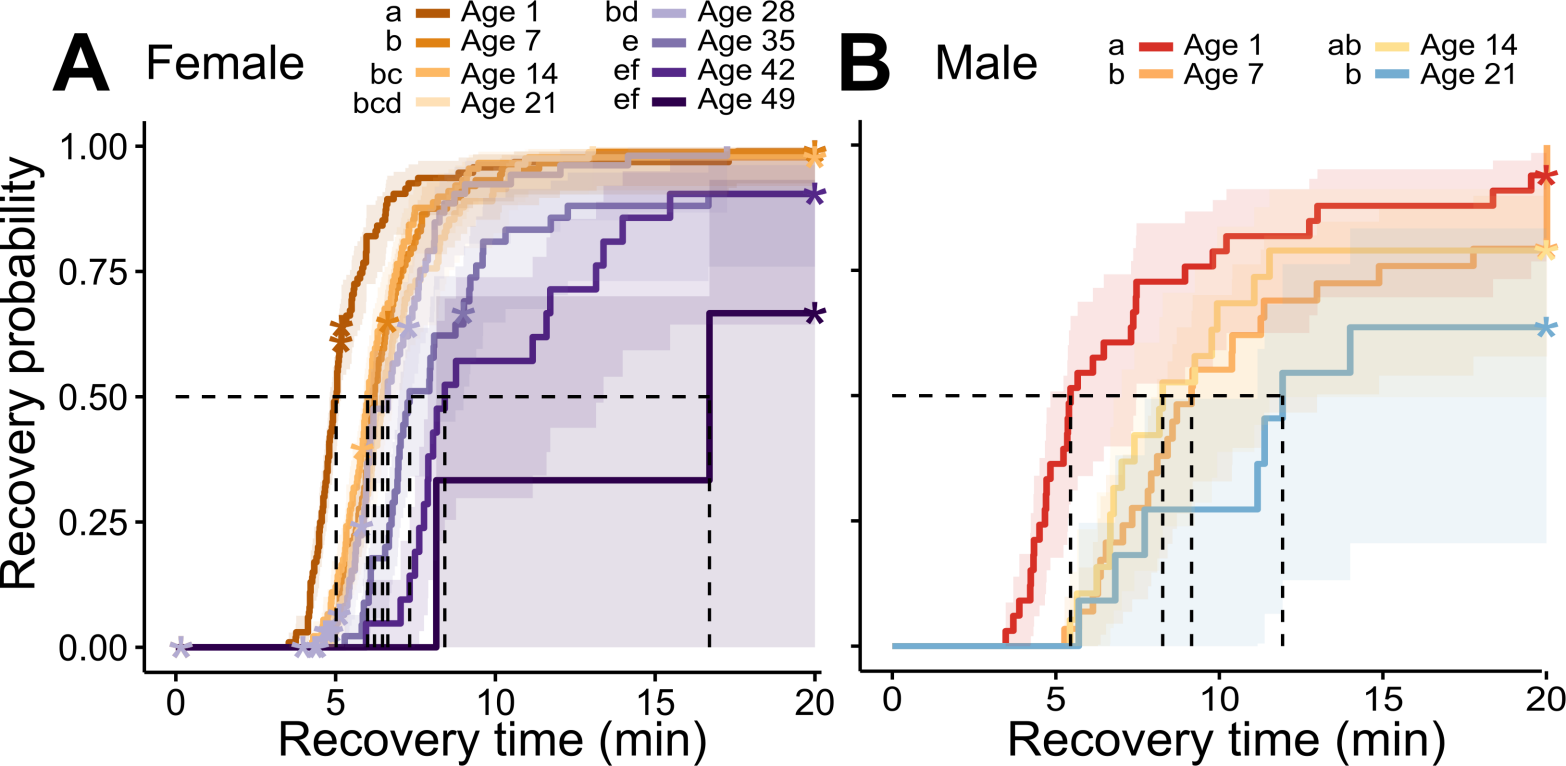
Temporal recovery curves of *Megachile rotundata* adult females (A) and males (B) exposed to weekly cold stress (0°C for 45 min) across their lifetime. Dotted lines mark the median chill-coma recovery times, shaded region shows the 95% CI, asterisks indicate censored individuals and lettering in legend shows significant differences (*p* < 0.05).

**Figure 3.**
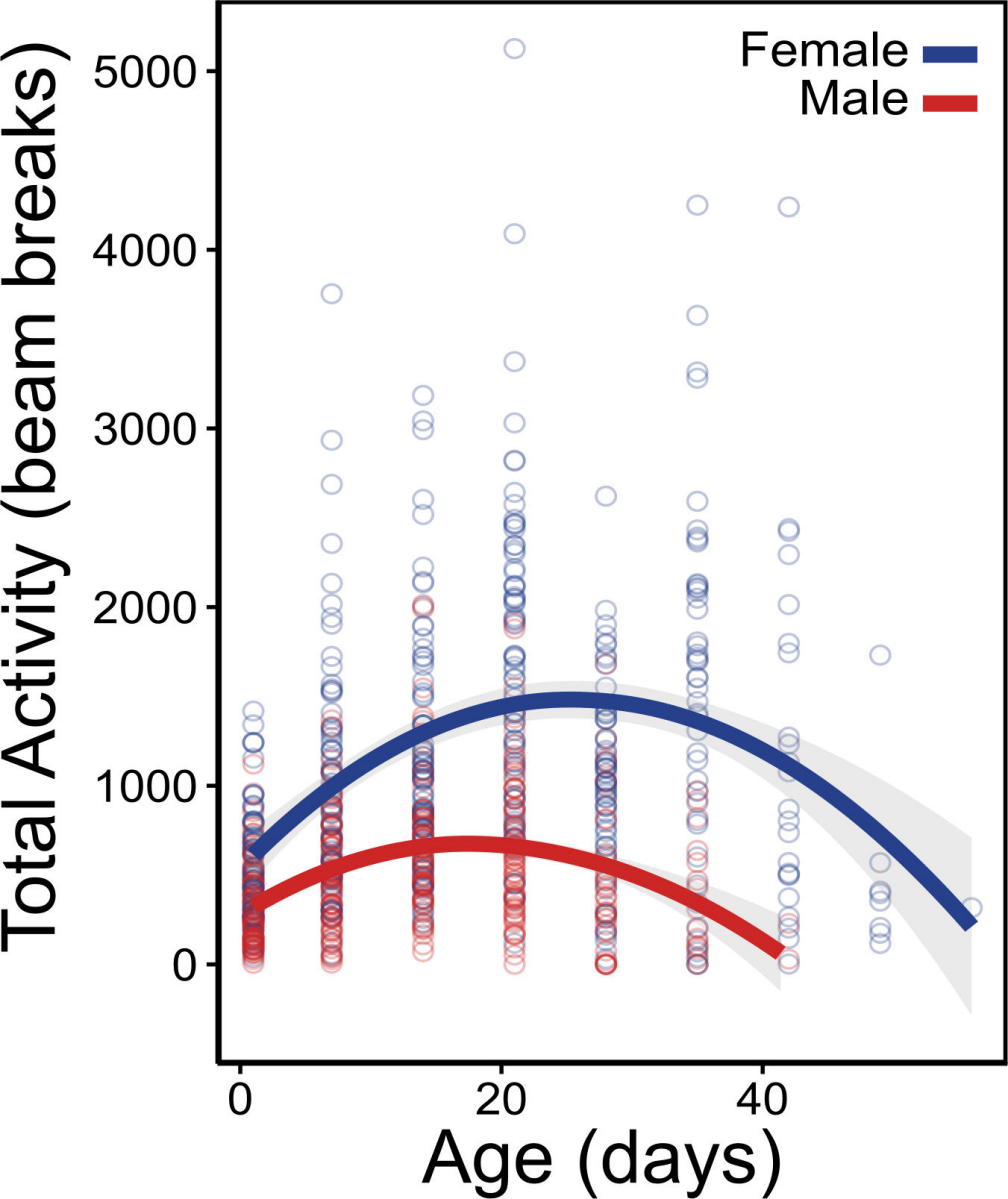
Locomotor activity across adult lifespan of *Megachile rotundata*. Total activity measured over 4 h during peak active time (10.00−14.00).

### (c) Cold tolerance declines with age

CCRTs were significantly different across sex and age (figure 2, table 2), with a significant interaction between age and lifespan for female bees (electronic supplementary material, table S1C). Recovery times were fastest for both sexes in newly emerged bees (Day 1), then plateaued for the following four weeks for females and two weeks for males, and then gradually increased with age. Best fit models included both individual ID and housing box as random effects (electronic supplementary material, table S1).

**Table 2.** Proportional hazard models for chill-coma recovery time across age in both female and male *Megachile rotundata* bees. HR, hazard ratio; CI, confidence interval.

age (days)	HR	95% **CI**	*p* value
**female**			
1	—	—	
7	0.47	0.35, 0.62	<0.001
14	0.53	0.40, 0.71	<0.001
21	0.4	0.30, 0.54	<0.001
28	0.39	0.28, 0.54	<0.001
35	0.22	0.15, 0.32	<0.001
42	0.17	0.10, 0.27	<0.001
49	0.09	0.02, 0.36	<0.001
**male**			
1	—	—	
7	0.45	0.26, 0.78	0.004
14	0.5	0.27, 0.94	0.03
21	0.29	0.13, 0.66	0.003

### (d) Locomotor activity declines with age

Locomotor activity was significantly different between sexes and followed a negative parabolic pattern with age (electronic supplementary material, table S1D; figure 3). Peak activity occurred at age 28 for females and 21 for males, after which activity begins to decline.

### (e) Lifespan and early-life performance

After characterizing how age affects performance, we explored whether long-lived and short-lived individuals had divergent performance trends during early life that may have predictive power on bee lifespan. All best fit models included individual ID as a random effect. Male bees showed no significant trends across experiments when comparing short-lived and long-lived bees and when assessing the relationship between early-life performance and lifespan (electronic supplementary material, table S2). Thus, results will focus on female bees, where short-lived bees lived up to 21 days and long-lived bees lived up to 49−56 days. When assessing short and long-lived bees, repeatability (i.e. proportion of variance explained by individual variation) for CCRT and locomotor activity increased by a factor of 2.6 and 2.5, respectively, compared with the whole experimental populations (electronic supplementary material, tables S1 and S3). Mass-corrected resting metabolic rates for short-lived and long-lived females were not significantly different (electronic supplementary material, table S3A, figure S4A), and there was no relationship between early-life resting metabolic rates and lifespan (figure S4B).

**Figure 4.**
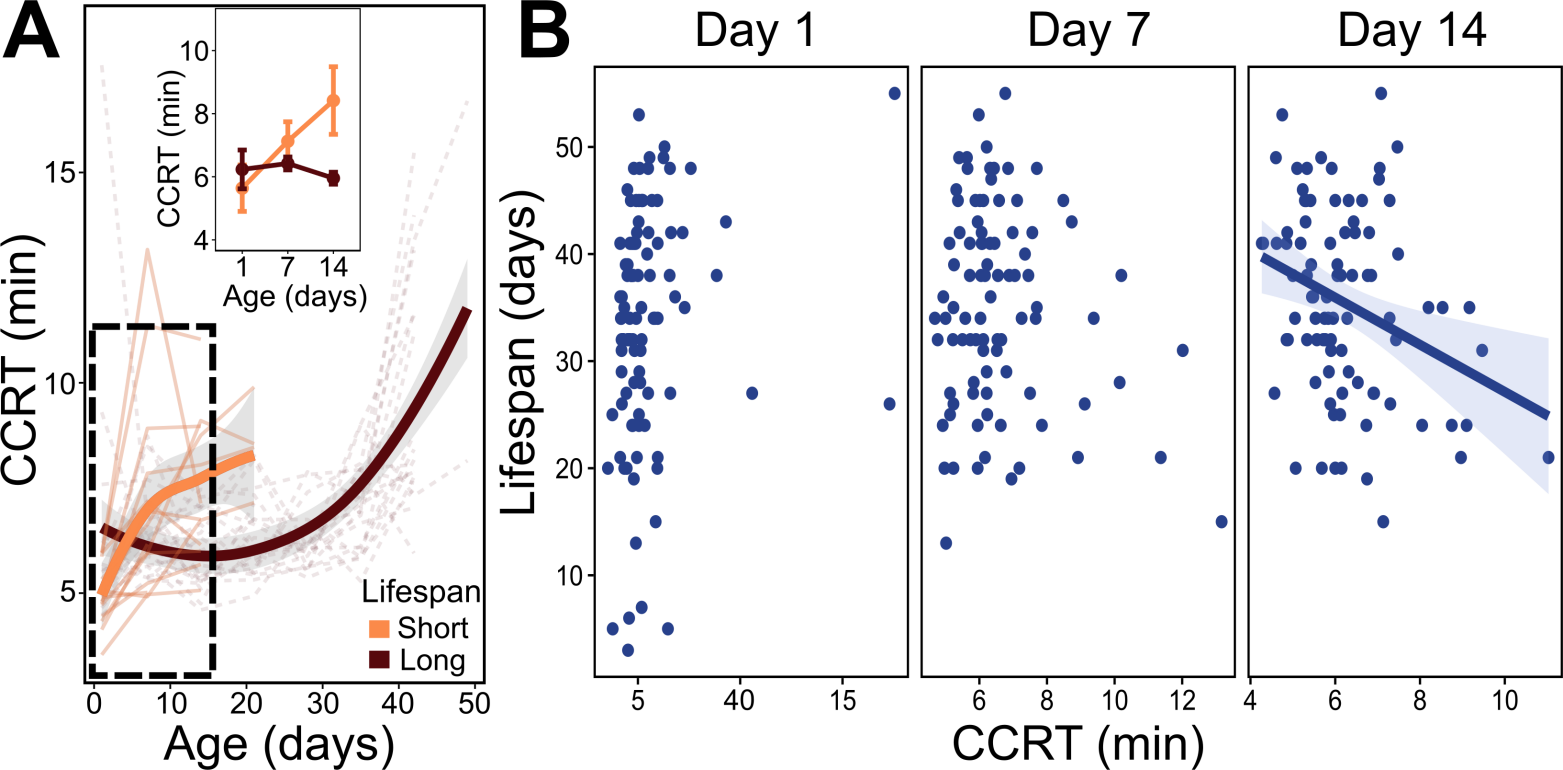
Chill-coma recovery time (CCRT) and longevity of *Megachile rotundata* female adults. (A) CCRT reaction norms of short-lived bees (orange) and long-lived bees (brown) across their lifetime. Thin lines represent individual CCRT, thick lines show mean and shaded area indicates 95% CI. Top inset shows analysis between early-life age groups (group delimited by dotted box). (B) Whole age-group population relationship between lifespan and early-life cold tolerance. Significant trends shown by a solid line and shaded area indicates 95% CI.

### (f) Cold tolerance and lifespan

CCRTs for short-lived and long-lived female bees were significantly different (age × Long.Lived interaction, conditional *R*^2^ = 0.44; electronic supplementary material, table S3B; figure 4A), where short-lived bees decline in cold tolerance and long-lived bees maintain fast recovery. Cold tolerance for the whole female population at Day 14 had a significant relationship in which lifespan declined as recovery time increased (*F*_1,86_= 8.89, adjusted *R*^2^ = 0.09, *p* = 0.003; figure 4B), albeit explaining only 9% of the variance on lifespan.

### (g) Locomotor activity lifespan

**Figure 5.**
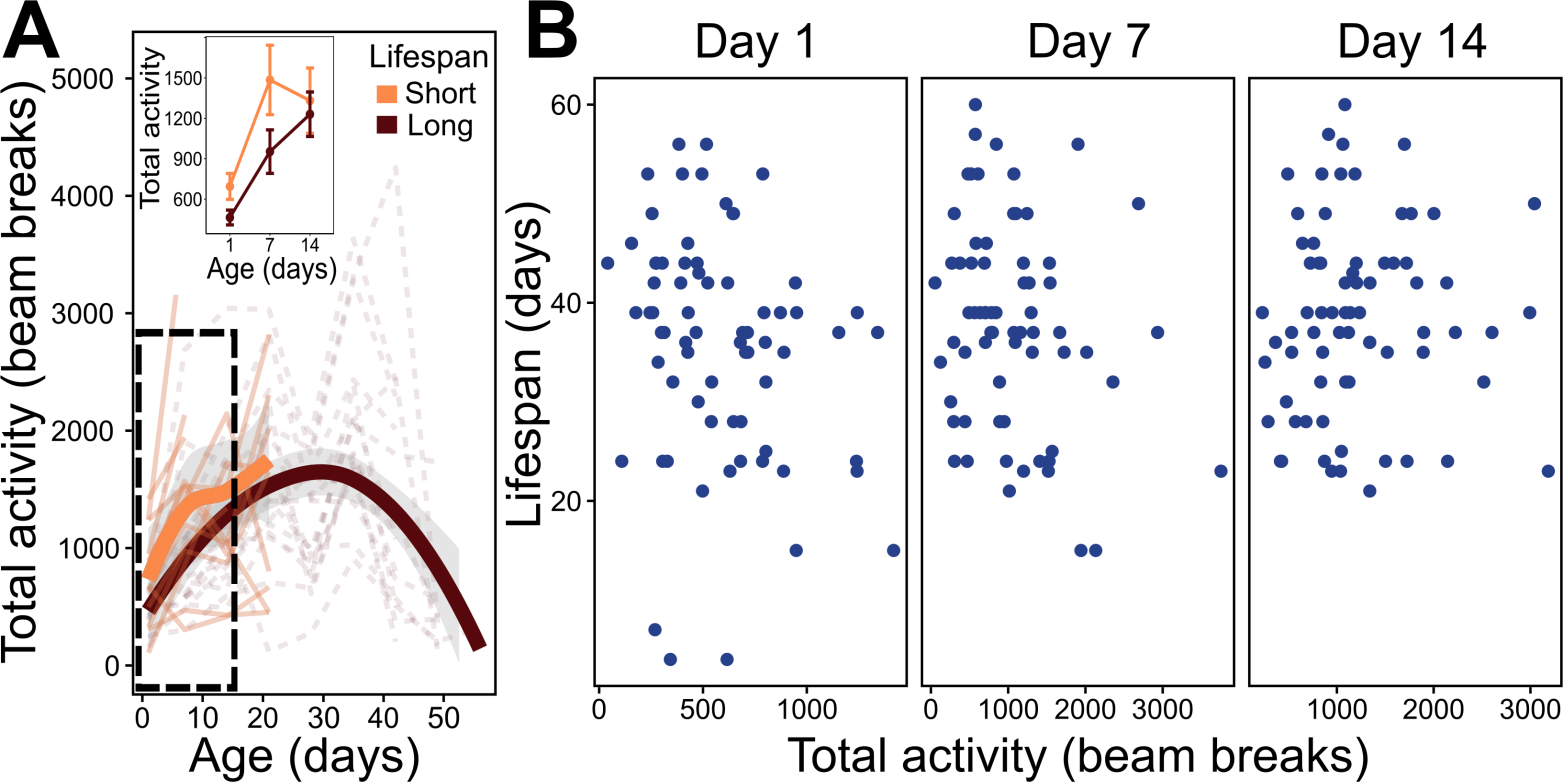
Locomotor activity and longevity of *Megachile rotundata* female adults. (A) Activity reaction norms of short-lived bees (orange) and long-lived bees (brown) across their lifetime. Thin lines represent individual activity, thick lines show mean and shaded area indicates 95% CI. Top inset shows analysis between early-life age groups (group delimited by dotted box). (B) Whole age-group population relationship between lifespan and early-life locomotor activity. Significant trends shown by a solid line and shaded area indicates 95% CI.

Early-life locomotor activity for short-lived and long-lived females was not significantly different, yet short-lived bees tended to have higher activity than long-lived bees (electronic supplementary material, table S3C; figure 5A). Population activity at Days 1 and 7 of emergence also had a near significant relationship to lifespan with high activity early in life associated with shorter lifespans (Age 1 *F*_1,68_ = 3.878, *p* = 0.053, Age 7 *F*_1,65_ = 3.913, *p* = 0.052; figure 5B).

### (h) Mass at emergence and lifespan

**Figure 6.**
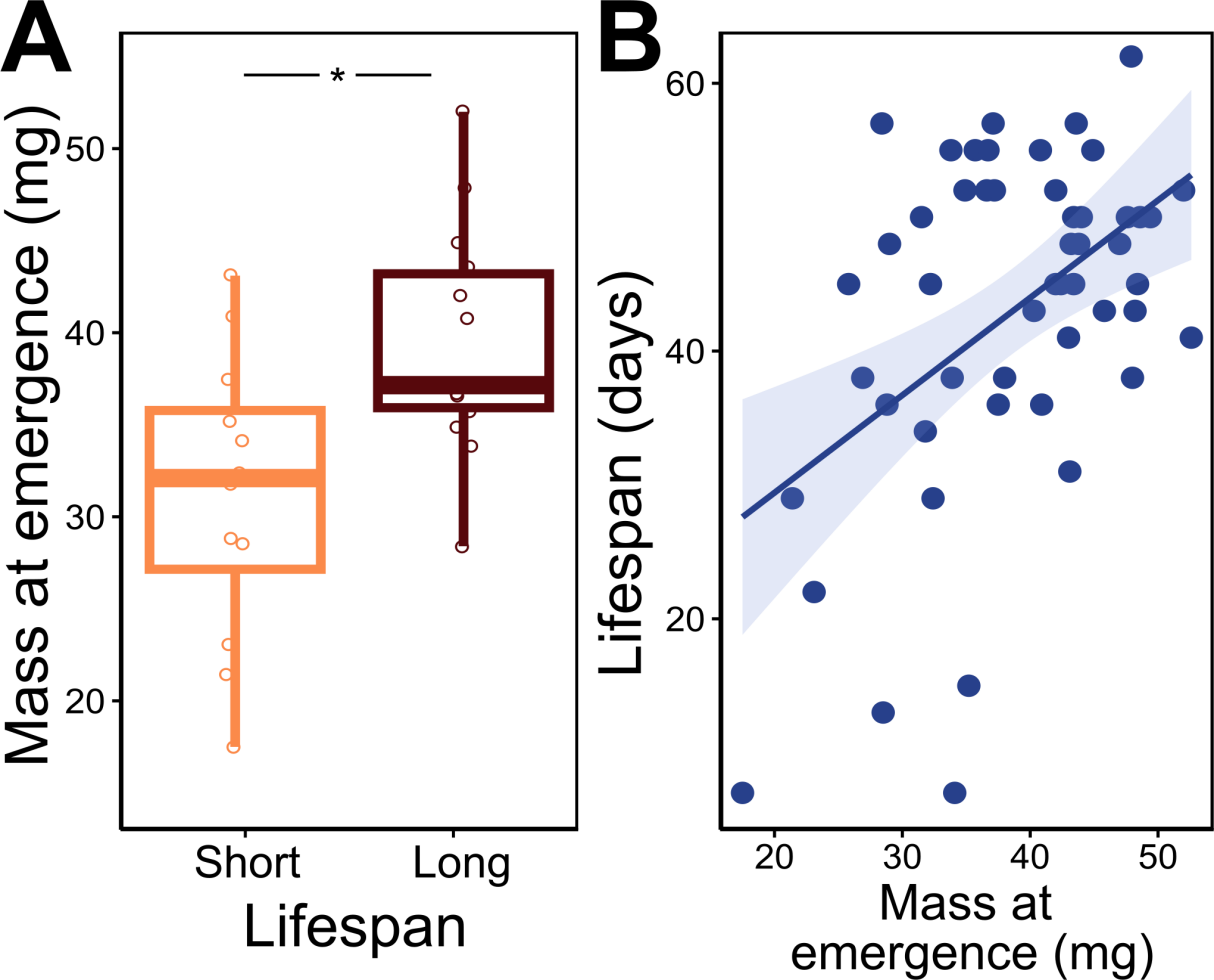
Mass at emergence and lifespan of *Megachile rotundata* adult females. (A) Comparison between short-lived and long-lived bees and mass at emergence (Day 1). (B) Emergence mass for whole female population and lifespan. Female mass was measured for the metabolic rate experiment individuals. Significant trend shown by the solid line and shaded area indicates 95% CI.

Throughout the metabolic rate experiments, mass was recorded every week, including the mass at emergence (Day 1). Female mass at emergence differed significantly between short-lived and long-lived bees (*F*_1,24_ = 8.983, *p* < 0.01; figure 6A), while male mass at emergence showed no relation to short- or long-lived bees (*F*_1,21_ = 0.232, *p* = 0.63). For the whole female population, mass at emergence also showed a significant relationship to lifespan, where lifespan increased on average by 7.28 days for every 10 mg of mass at emergence (*F*_1,49_ = 14.08, *p* < 0.001; figure 6B).

## 4. Discussion

Variation in performance within the same-age groups may help explain why organisms age at different rates. Here we conducted a longitudinal study testing how three different performances (locomotor activity, cold tolerance and resting metabolic rates) change with age in adults of the solitary bee *M. rotundata* and assessed whether early-life performance is associated with individuals’ lifespan.

### (a) Performance trends and ageing

Cold tolerance, measured as CCRT, declined with age for both females and males, showing a nonlinear trend, with the highest tolerance in young adults and lowest tolerance in old adults. High-stress tolerance in young adults is also seen in other insect systems and likely due to carry-over tolerance from the previous life stage [22], in this case a sessile pupa. The decline in cold tolerance with age matches past studies assessing chill-coma recovery and other thermal stress studies [23]. Our results show a period of several weeks where both females and males maintain a robust cold tolerance before performance begins to decline. This trend suggests that the mechanisms for maintaining cold tolerance slowly degrade with age. Although substantial work has been done understanding the mechanisms of chill-coma recovery and regaining ion-balance [24,25], little is known about the mechanisms underlying the loss of cold tolerance with ageing. For example, certain heat-shock proteins that are readily upregulated under thermal stress and associated with cold tolerance rapidly decline with age in *Drosophila melanogaster* [8]. A recent study also shows senescence in the renal system (Malpighian tubules) of *Drosophila* [26], posing a tentative mechanism for the decline in the ability to recover effectively later in age. Also, sex differences in aging rates of organs [27] may help explain the rapid decline seen in males compared with female bees. Our results lay a robust foundation to test these new hypotheses on how cold stress tolerance declines with age and explore the physiological mechanisms that underlie the loss of function during senescence.

Populations of the solitary bee used in this study occur at high latitudes with highly seasonal environments [28]. Our results suggest that during the last weeks of the growing season and towards the seasonal transition into autumn, early cold events may disproportionally affect older individuals, leading to shifts in population structure. Finally, highlighting the sex differences seen in cold tolerance in this solitary bee, repeated cold stress significantly reduces male median lifespan by almost 50% without eliciting mass mortality. Interestingly, *D. melanogaster* females repeatedly exposed to cold showed no effect on short-term mortality but instead incurred a reproductive trade-off compared with a single long cold-exposed treatment [29]. Whether this reproductive cost is due to a shortening of lifespan remains an exciting open question, especially in the context of resource allocation under stressful environments [30]. Similar sex differences in stress tolerance were also seen in *D. melanogaster* after a single short heat stress treatment [21], and in age-specific CCRT between males and females [31]. This adds to a growing number of studies showing sexual dimorphism in thermal stress tolerance [32–34]. With increasing frequency and intensity of extreme events such as heat waves and cold snaps, thermal stress may act as selective force for one sex but not the other. Including these sex-specific differences in stress tolerance in population models could greatly enhance the predictive power of future winners and losers with ongoing climate change.

Locomotor activity declined during old age in both sexes. The activity pattern followed a negative parabolic trend, where young adults gradually increase locomotor activity, reaching a peak in activity during middle age, followed by a gradual decline. Our results show similar trends to a recent study using the same bee species [18] and across other species [35,36] suggesting that locomotor activity is likely a good baseline performance metric to study senescence across insects. Similar to cold tolerance, locomotor activity showed a clear sex-specific pattern, with males having generally lower activity than females. These results potentially mirror the behavioural differences seen between males and females, where females must conduct high-performance tasks such as nest building and food provisioning, while males only forage and find mates. It is important to note that the repeated measures of locomotor activity slightly reduced lifespan for both males and females (figure 1). Because activity trials were conducted during the daily peak active hours for this bee species, we hypothesize that the reduced lifespan may be due to a weekly short exposure to diet restriction. However, if this is the case, it is intriguing that a recurring 4 h treatment without food followed by a week of recovery would have been stressful enough to reduce median lifespan by 15% in males and 22% in females.

Finally, resting metabolic rates and mass remained strikingly constant across adult bee lifespan, not supporting our initial hypothesis. Yet, our results match observations seen in several species like *Drosophila* [37], a seabird [38] and some butterflies [39,40]. Our overall results show that, from the traits measured, only performances that require energy declined with age (locomotor activity and chill-coma recovery [17,41,42]), while resting metabolic rates and mass are maintained strikingly unchanged. Decoupling traits that decline with age from traits that remain unaffected with age can provide insights into the dynamics of aerobic scope during senescence. For example, from an energy budget perspective, reallocating energy towards maintenance processes at the expense of other performances, such as stress tolerance in old age, may reveal evidence of a resource-based trade-off during senescence. Alternatively, costly performances such as stress tolerance may simply become too energetically costly at later ages, leading to a decline in performance while maintaining baseline energy use. Finally, it is important to consider that in this study individuals were not able to fly, or reproduce, which can potentially mask energetic pool utilization. However, we predict that more energy-demanding performances like flight would likely exacerbate, and not attenuate, energetic constraints with age [43].

### (b) Early-life performance and lifespan

First, it is noteworthy that male bees showed no significant trends with early-life performance and lifespan across any traits tested in this study. It is difficult to conclude whether these results are due to differences in the scale of lifespan compared with females (i.e. one week for males comprises over 20% of their median lifespan, while it is under 15% for female bees) or whether the performances tested are simply poor predictors of male lifespan and may not be under the same selective pressures as female bees. Hereafter, we discuss the early life and ageing patterns seen among female bees. We encourage future studies to explore whether other performance measures may be better predictors of lifespan for males.

In this study, we tested whether short and long-lived bees differed in early-life performance and whether lifespan correlates with performance within early-life age groups. When comparing short- and long-lived bees, our results show some differences between lifespan groups. When assessing lifespan and early-life performance, we find support for cold tolerance and mass at emergence but not for locomotor activity and resting metabolic rates. The slowest bees to recover from cold at Day 14 tended to have short lifespans, supporting our hypothesis that cold tolerance has some predictive power on lifespan. These results also support the notion that metabolic robustness in cold tolerance can translate into other fitness and life-history traits. We suggest that the slow recovery response seen in short-lived individuals may be due to a rapid degradation of the metabolic network required for fast recovery compared with long-lived bees.

Mass at emergence showed a strong correlation with female lifespan. Evidence suggests that body condition (e.g. body size and mass) can be associated with high survival and lifetime reproduction, especially in seasonal environments [44–46]. The link between mass at emergence and lifespan highlights important trends with ongoing climate change [47], especially for organisms with similar life histories to the solitary bees. For example, most solitary bees build nests made of multiple brood cells, each containing a single egg and a set amount of pollen provisions [48]. The pollen provisions are enough resources for the offspring to complete development into the next generation of adult bees; therefore, the amount of food provisions determines the size of the resulting offspring [49,50]. Thus, the body condition of emerging new adult bees is essentially the direct result of their mother’s investment into their brood cells. Under non-stressful environmental conditions, mass at emergence is a measure of the mother’s fitness investment and may serve as an indicator of population health. Small adults have been documented in wild populations under poor foraging conditions [51,52]. We encourage field studies to explore the relationship between mass at emergence and lifespan, as it may offer a key parameter for population models assessing the effects of changing and unpredictable environments [30].

## 5. Conclusion

Overall, this study shows that, even with substantial individual-level variation in lifespans and performance, there are clear links between performance, age and sex in the solitary bee *M. rotundata*. We show that locomotion and cold tolerance gradually decline in older individuals but not resting metabolic rates and mass. This observation adds to the growing evidence that not all functional traits senesce [4,53]. However, variation in performance within the same age groups and within individuals across time likely holds physiological and/or genetic underpinnings that remain underexplored. One objective of this study was to determine whether early-life performance could inform lifespan. We found clear trends for cold tolerance and mass at emergence that can be used in future studies on determining biological age, especially when requiring non-destructive methods to separate potentially long- and short-lived individuals. Finally, our results provide empirical evidence that physiologically robust individuals are more likely to live longer, and show that sex differences in physiological traits can have profound effects that modulate lifespan, and must be taken into account when studying ageing.

## Data Availability

Data files are provided in the Dryad data repository [54]. Supplementary material is available online [55].
